# Blood-based cerebral biomarkers in preeclampsia: Plasma concentrations of NfL, tau, S100B and NSE during pregnancy in women who later develop preeclampsia - A nested case control study

**DOI:** 10.1371/journal.pone.0196025

**Published:** 2018-05-02

**Authors:** Lina Bergman, Henrik Zetterberg, Helena Kaihola, Henrik Hagberg, Kaj Blennow, Helena Åkerud

**Affiliations:** 1 Department for Women’s and Children’s health, Uppsala University, Uppsala, Sweden; 2 Center for Clinical Research, Falun, Sweden; 3 Institute of Neuroscience and Physiology, Department of Psychiatry and Neurochemistry, Sahlgrenska Academy, University of Gothenburg, Mölndal, Sweden; 4 Clinical Neurochemistry Laboratory, Sahlgrenska University Hospital, Mölndal, Sweden; 5 UCL Institute of Neurology, Queen Square, London, United Kingdom; 6 UK Dementia Research Institute, London, United Kingdom; 7 Department of Immunology, Genetics and Pathology, Uppsala University, Uppsala, Sweden; 8 Perinatal Center, Department of Obstetrics and Gynecology, Sahlgrenska Academy, University of Gothenburg, Gothenburg, Sweden; 9 Centre for the Developing Brain, King's College, London, United Kingdom; National Research Council of Italy, ITALY

## Abstract

**Objective:**

To evaluate if concentrations of the neuronal proteins neurofilament light chain and tau are changed in women developing preeclampsia and to evaluate the ability of a combination of neurofilament light chain, tau, S100B and neuron specific enolase in identifying neurologic impairment before diagnosis of preeclampsia.

**Methods:**

A nested case-control study within a longitudinal study cohort was performed. 469 healthy pregnant women were enrolled between 2004–2007 and plasma samples were collected at gestational weeks 10, 25, 28, 33 and 37. Plasma concentrations of tau and neurofilament light chain were analyzed in 16 women who eventually developed preeclampsia and 36 controls throughout pregnancy with single molecule array (Simoa) method and compared within and between groups. S100B and NSE had been analyzed previously in the same study population. A statistical model with receiving characteristic operation curve was constructed with the four biomarkers combined.

**Results:**

Plasma concentrations of neurofilament light chain were significantly increased in women who developed preeclampsia in gestational week 33 (11.85 ng/L, IQR 7.48–39.93 *vs* 6.80 ng/L, IQR 5.65–11.40) and 37 (22.15 ng/L, IQR 10.93–35.30 *vs* 8.40 ng/L, IQR 6.40–14.30) and for tau in gestational week 37 (4.33 ng/L, IQR 3.97–12.83 *vs* 3.77 ng/L, IQR 1.91–5.25) in contrast to healthy controls. A combined model for preeclampsia with tau, neurofilament light chain, S100B and neuron specific enolase in gestational week 25 displayed an area under the curve of 0.77, in week 28 it was 0.75, in week 33 it was 0.89 and in week 37 it was 0.83. Median week for diagnosis of preeclampsia was at 38 weeks of gestation.

**Conclusion:**

Concentrations of both tau and neurofilament light chain are increased in the end of pregnancy in women developing preeclampsia in contrast to healthy pregnancies. Cerebral biomarkers might reflect cerebral involvement before onset of disease.

## Introduction

Cerebral complications in preeclampsia are common causes for mortality but are hard to predict.[1, 2] Some women with preeclampsia and a majority of women with eclampsia exhibit cerebral oedema known as Posterior Reversible Encephalopathy Syndrome (PRES), secondary to a compromised blood brain barrier and hyperperfusion.[3] Clinical symptoms and blood pressure provide poor predictive values for PRES and eclampsia.[4] Early cerebral involvement in women developing preeclampsia is poorly understood and most research focuses on cerebrovascular function in women already diagnosed with preeclampsia.[5] These studies are often limited to retrospective cohort- or animal studies.[6]

Neurofilament light chain (NfL) and tau are neuronal proteins produced in brain tissue, but mRNA has also been found to a very small extent in extracerebral tissue.[7–10]

Plasma concentrations of S100B and neuron-specific enolase (NSE) are increased in women developing preeclampsia from gestational week 33 and S100B correlates to presence of neurological symptoms in women with preeclampsia.[11–13] There are no previous human studies on the cerebral biomarkers tau and NfL in peripheral blood in pregnancy.

Cerebral biomarkers are promising in combinations to predict adverse outcome in traumatic brain injury[14] but has never been investigated in women with preeclampsia.

Ultra-sensitive Single molecule array (Simoa), has emerged for the measurement of very low concentrations of tau and NfL in plasma and serum.[15, 16] This method has now also been used to measure the proteins in our cohort of women.

The primary aim in this study was to evaluate if concentrations of NfL and tau are changed in women developing preeclampsia. The secondary aim was to evaluate the potential cerebral involvement in women with preeclampsia before onset of disease in a combination model with peripheral concentrations of NfL, tau, S100B and NSE.

The null hypothesis was that there would be no difference between women developing preeclampsia and women with normal pregnancies regarding plasma concentrations of NfL and tau.

## Materials and methods

The study was approved by the regional ethics committee at Uppsala University (Ethics committee reference number 2005:75, date of approval April 6, 2005). Informed consent was obtained from each woman included in the study.

### Population

The population is described and published earlier.[11, 12] A power calculation was not possible to perform since the concentrations of the biomarkers in women with preeclampsia are unknown. A cohort of healthy women (n = 469) was enrolled between gestational week 8–12 at five centres in Värmland, Sweden, during 2004–2007. Preeclampsia was defined as hypertension (>140/90 mm Hg at two different occasions > 6 hours apart) and proteinuria (>300 mg/24 hours or >2+ on a dipstick) after 20 weeks of gestation. Exclusion criteria were chronic hypertension, upper urinary tract infection, pre-existing renal disease or diabetes mellitus. Plasma samples were collected at gestational weeks 10, 25, 28, 33 and 37 at the routine visit to antenatal care. 20 women developed preeclampsia and 16 women had blood samples available for analysis. A nested case control study was performed where 36 women from the population with healthy pregnancies were randomly selected as controls. The same cases and controls that were used for earlier publications were also used in this study, resulting in the number of 36 controls with available samples.

### Sample collection

Blood samples were collected in Li/Hep tubes and after centrifugation that occurred within 30 minutes, plasma was frozen in cryotubes at -70°C until analyzed. Mean storage time until analyze was four years for S100B, seven years for NSE and nine years for NfL and tau. All samples were frozen in aliquots after a maximum time of 60 minutes, handled according to the same routine and thawed only for analysis of S100B, NSE, NfL or tau.

### Electrochemiluminescence immunoassay and Simoa

The plasma NfL concentration was measured using an in house Single molecule array (Simoa) method as previously described in detail.[17] The plasma tau concentration was measured using the Human Total Tau 2.0 kit and the Simoa platform (Quanterix, Lexington, MA) as previously described in detail.[18] All measurements were performed in one round of experiments using one batch of reagents by board-certified laboratory technicians who were blinded to clinical data. Intra-assay coefficients of variation were 11–12% for NfL and 7.4–8.4% for tau.

### Enzyme linked immunosorbent assay

Plasma S100B and NSE concentrations were measured by ELISA and published previously in the same population.[11, 12] The samples were run according to the manufacturer’s recommendation and the intra and inter assay coefficient of variation were 4.6 and 2.8% and 4.3 and 3.1% respectively for S100B and NSE.

### Statistics

Demographics and clinical characteristics were compared between groups by student’s t-test for continuous variables and chi square test for proportions. Variables are presented as means with standard deviations.

For the analyses of NfL and tau, logarithmic values were used to obtain normal distribution. For comparisons between groups, student t-test was used. For comparisons over time within groups, paired t-test was used with Bonferroni correction. Values are presented as medians with interquartile range (IQR).

Receiver operator characteristics (ROC) curves were constructed to determine predictive ability for preeclampsia in different gestational weeks.

All statistical tests were 2-tailed. A p-value <0.05 was considered statistically significant. Statistical analysis was performed using SPSS version 23.0 (IBM SPSS statistics, Armonk, New York) for MAC software package.

## Results

### Background characteristics

Maternal characteristics regarding demographics are presented in Table 1. Women developing preeclampsia did not differ from women with healthy pregnancies concerning age, parity, smoking status, body mass index (BMI), blood pressure at enrolment or infant birth weight. Women who developed preeclampsia had higher blood pressure at delivery, a shorter gestational length and more often used blood pressure medication. Four women developed early onset preeclampsia (diagnosis ≤34 weeks of gestation). One woman was delivered <34 weeks of gestation due to a placental abruption. All other women developed preeclampsia without end organ involvement. Median gestational age at diagnosis of preeclampsia was at 270 days (38.4 weeks). The earliest diagnosis was at 212 days (30 weeks) of gestation.

**Table 1:** Background characteristics of the population.

	Controls	Preeclampsia	p-value
	(n = 36)	(n = 16)	
Maternal age (years)	30 ± 4	29 ± 4	0.36
Nulliparous n (%)	21 (60)	13 (81)	0.11
Smokers n (%)	0	0	
BMI in first trimester (kg/m2)	24 ± 4	25 ± 5	0.23
*BP in first trimester (mmHg)*			
Systolic	111 ± 10	115 ± 11	0.25
Diastolic	65 ± 8	68 ± 9	0.26
MAP	80 ± 8	84 ± 8	0.20
Gestational age at diagnosis (days)		270 (246.5–283.5)	
BP medication n (%)	0 (0)	6 (38)	<0.001
Systolic	111 ± 10	115 ± 11	0.25
*At delivery*			
*BP (mm Hg)*			
Systolic	122 ± 14	150 ± 16	<0.001
Diastolic	72 ± 10	98 ± 7	<0.001
MAP	89 ± 10	116 ± 9	<0.001
Gestational length at delivery (weeks)	40.5 ± 1	39.0 ± 3	<0.01
Infant birth weight (g)	3714 ± 375	3587 ± 851	0.59
BP medication n (%)	0 (0)	6 (38)	<0.001

Values are presented as mean ± standard deviation and medians with 25^th^-75^th^ percentile as appropriate and categorical values as numbers (percentage); Student’s t-test; n, number; BMI, body mass index in early pregnancy; BP, blood pressure; MAP, mean arterial pressure

Blood samples from cases and controls have been analyzed for other biomarkers and thus some samples in different gestational weeks were no longer available at the time for this study. This is described in detail in the legends of Tables 2 and 3.

**Table 2:** Concentrations of NfL in plasma during pregnancy in women developing preeclampsia compared to women with normal pregnancies.

Gestational week	Controls (n = 36)	Preeclampsia (n = 16)
10	6.55 (3.93–8.20)	6.80 (4.67–8.53)
25	6.80 (3.70–9.25)	7.30 (5.10–10.65)
28	5.90 (4.15–8.80)	8.15 (6.48–19.90)
33	6.80 (5.65–11.40)*	11.85 (7.48–39.93)*
37	8.40 (6.40–14.30)**	22.15 (10.93–35.30)**

*p<0.05.

**p<0.01.

Values are presented as medians (ng/L) with 25^th^-75^th^ percentile. Statistical analyses were done by students t–test with logarithmic values for normal distribution.

In week 10, 30 controls and 12 preeclampsia patients had samples available. In week 25, the numbers were 30 controls and 9 preeclampsia. In week 28, the numbers were 29 controls and 10 preeclampsia. In week 33, the numbers were 30 controls and 6 preeclampsia. In week 37 the numbers were 26 controls and 8 preeclampsia.

**Table 3:** Concentrations of tau in plasma during pregnancy in women developing preeclampsia compared to women with normal pregnancies.

Gestational week	Controls (n = 36)	Preeclampsia (n = 16)
10	6.24 (4.70–8.42)	5.70 (3.90–8.12)
25	4.25 (3.15–6.94)	3.82 (2.54–4.16)
28	3.84 (3.11–5.46)	2.73 (2.26–5.06)
33	3.32 (2.38–5.45)	4.45 (2.09–5.24)
37	3.77 (1.91–5.25)*	4.33 (3.97–12.83)*

*p<0.05.

Values are presented as medians (ng/L) with 25^th^-75^th^ percentile. Statistical analyses were done by students t–test with logarithmic values for normal distribution.

In week 10, 28 controls and 12 preeclampsia patients had samples available. In week 25, the numbers were 30 controls and 9 preeclampsia. In week 28, the numbers were 30 controls and 9 preeclampsia. In week 33, the numbers were 29 controls and 7 preeclampsia. In week 37 the numbers were 25 controls and 9 preeclampsia.

### Plasma levels of NfL during pregnancy

**Fig 1:**
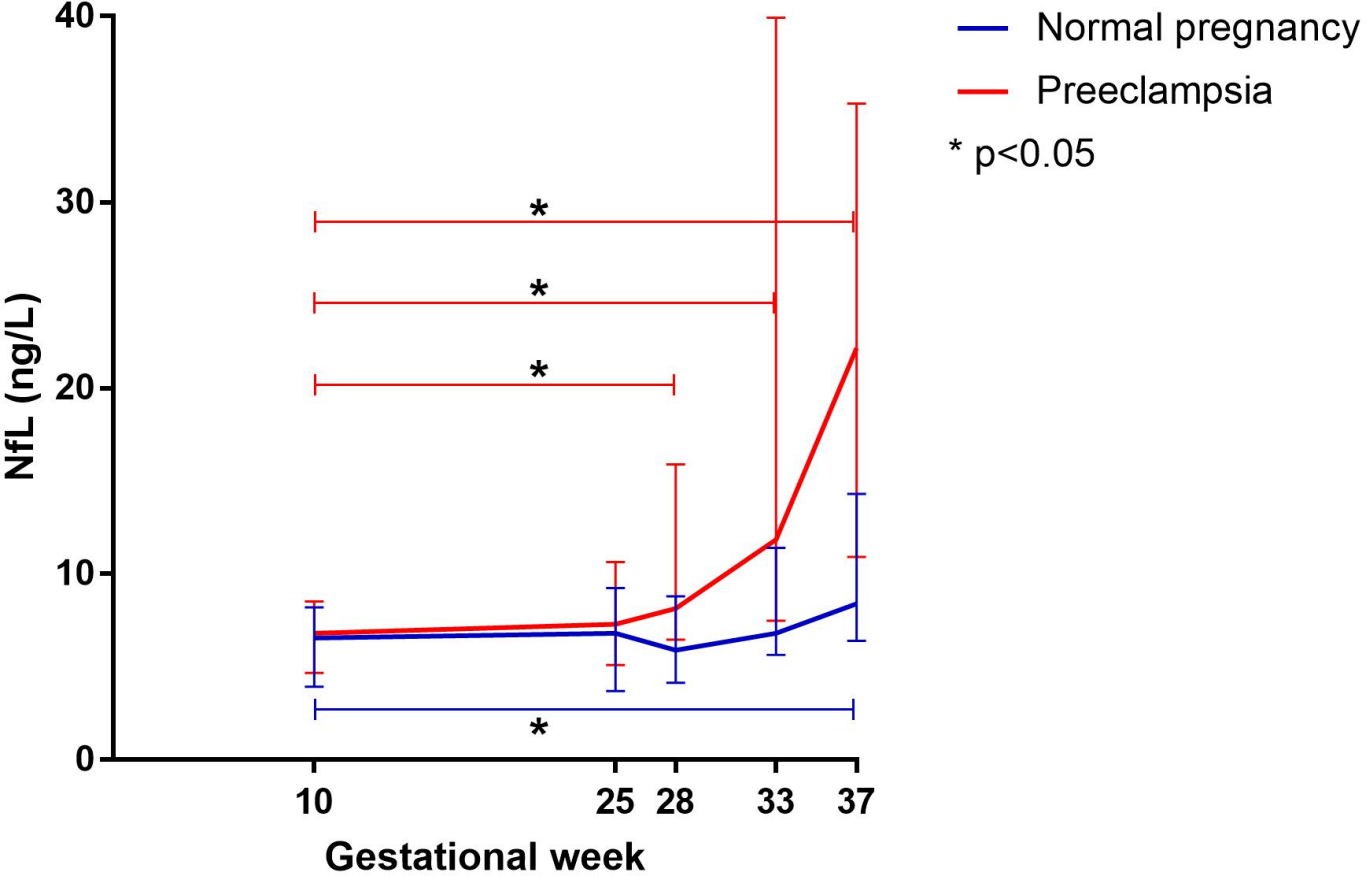
Plasma levels of neurofilament light chain (NfL) during pregnancy in normal pregnancies and those developing preeclampsia. Line diagram showing median concentrations with interquartile range (IQR) of NfL during pregnancy in women developing preeclampsia (red line) and in women with normal pregnancies (blue line). In women developing preeclampsia, concentrations of NfL were increased in gestational week 28, 33 and 37 compared to gestational week 10 (p<0.05). In healthy pregnancies, concentrations of NfL increased in gestational week 37 compared to gestational week 10 (p<0.05). Women developing preeclampsia had increased concentrations of NfL in gestational weeks 33 and 37 in contrast to women with healthy pregnancies (p<0.05).

In women developing preeclampsia, there was no difference in plasma concentrations of NfL in gestational week 25 compared to gestational week 10. There was a significant increase in plasma NfL concentrations in gestational week 28 (p<0.05), 33 (p<0.05) and 37 (p<0.05) compared to gestational week 10. (Fig 1)

In normal pregnancy, there was no difference in plasma concentrations of NfL in gestational week 25, 28 and 33 compared to gestational week 10, but there was a significant increase in gestational week 37 (p<0.05). (Fig 1)

Between groups, plasma concentrations of NfL were similar in gestational weeks 25 and 28 compared to gestational week 10, but women who developed preeclampsia had higher plasma concentrations of NfL in gestational weeks 33 (p<0.05) and 37 (p<0.01) compared to women with normal pregnancies. (Table 2)

There was a tendency to increased concentrations of NfL in early onset preeclampsia compared to late onset preeclampsia in gestational week 33 (24.5, IQR 13.4–64.9 vs 9.15, IQR 6.43–26.28 ng/L, p = 0.15) but the numbers were small and the difference was not statistically significant.

### Plasma levels of tau during pregnancy

**Fig 2:**
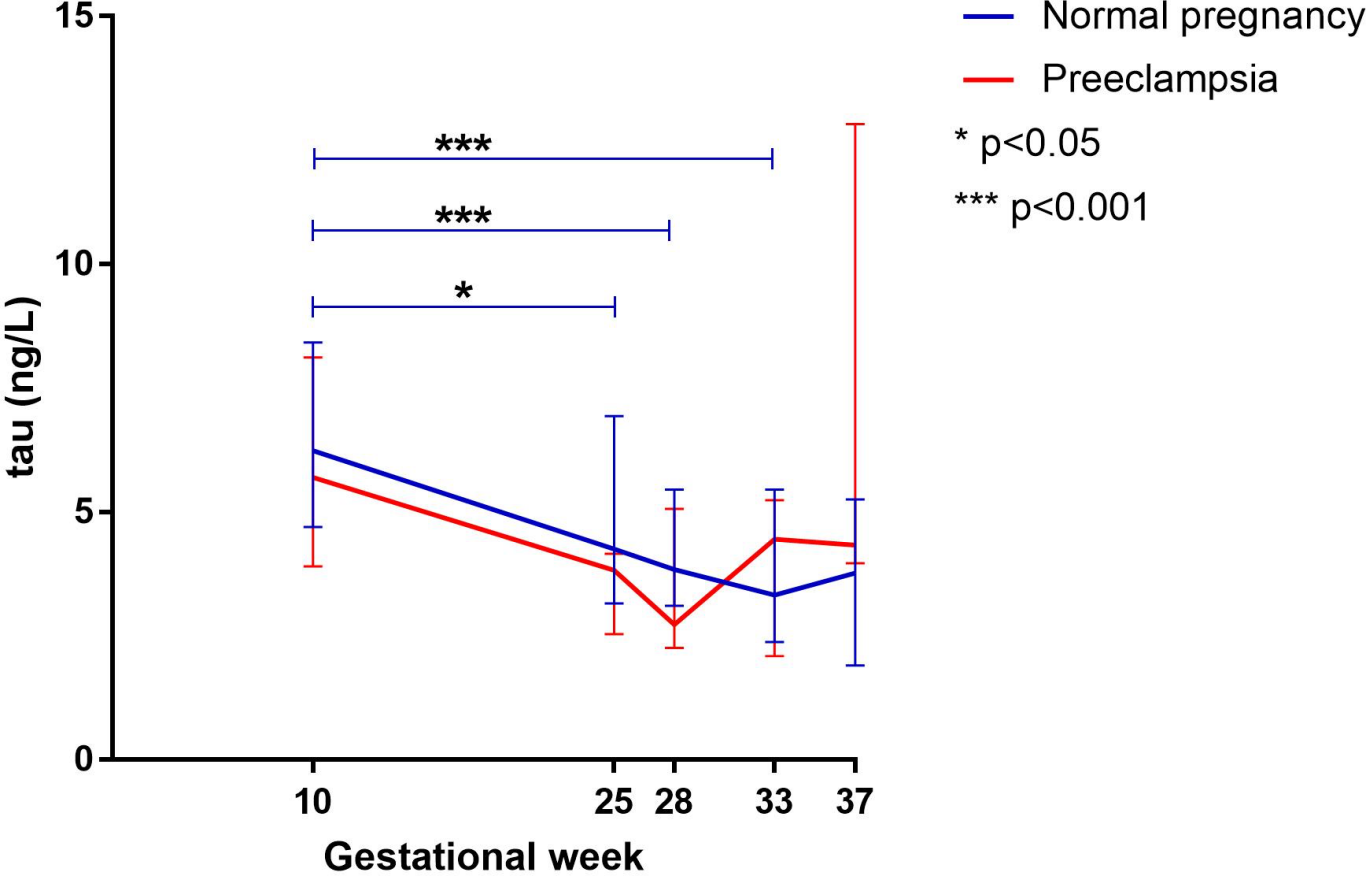
Plasma levels of tau during pregnancy in normal pregnancies and those developing preeclampsia. Line diagram showing median concentrations with interquartile range (IQR) of tau during pregnancy in women developing preeclampsia (red line) and in women with normal pregnancies (blue line). In women developing preeclampsia, concentrations of tau remained unchanged throughout pregnancy. In healthy pregnancies, concentrations of tau decreased in gestational week 25, 28 and 33 (p<0.05) and showed a borderline significant reduction in gestational week 37 compared to gestational week 10 (p = 0.06). Women developing preeclampsia had increased levels of tau in gestational week 37 in contrast to women with healthy pregnancies (p<0.05).

In women developing preeclampsia, there was no difference in plasma concentrations of tau throughout pregnancy. (Fig 2)

In normal pregnancy, there was a significant reduction in plasma concentrations of tau in gestational week 25 (p<0.05), 28 (p<0.001) and 33 (p<0.001) compared to gestational week 10. There was a borderline significant reduction in plasma concentrations of tau between gestational week 10 and 37 (p = 0.06). (Fig 2)

Between groups, there were no statistical significant differences in plasma concentrations of tau between groups in gestational weeks 10, 25, 28 or 33. In gestational week 37, women with preeclampsia experienced higher plasma concentrations of tau than women with normal pregnancies (p<0.05). (Table 3)

#### Combination of cerebral biomarkers for preeclampsia

Combination models with ROC curves were constructed with NSE, S100B, tau and NfL as indicators for cerebral involvement in preeclampsia in gestational week 10, 25, 28, 33 and 37.

**Fig 3:**
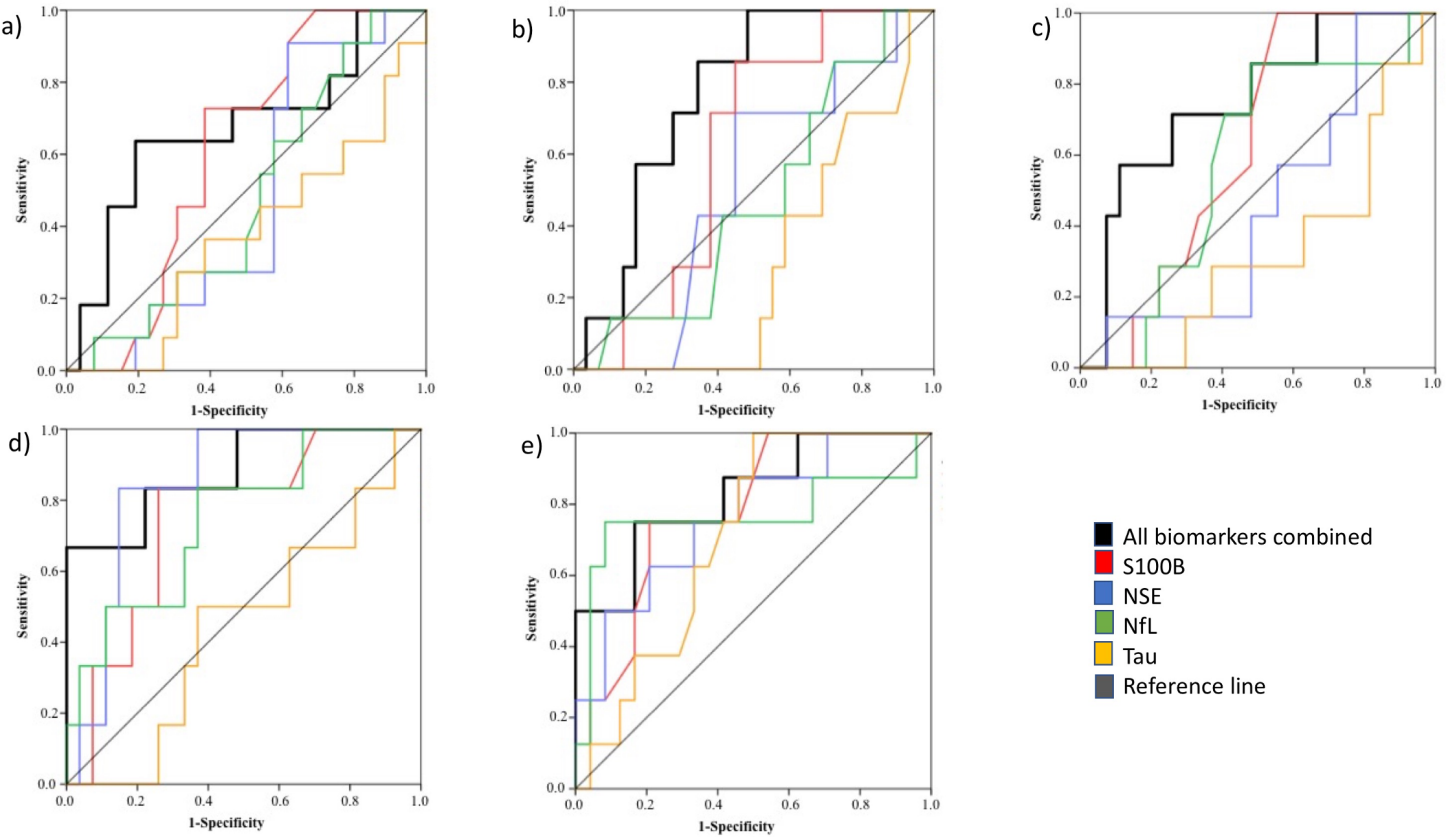
Receiver Operating Characteristic (ROC) curves using S100B, Neuron Specific Enolase (NSE), Nerofilament Light chain (NfL) and tau combined for determining preeclampsia at specific gestational weeks. a) 10, Area Under the Curve (AUC) = 0.67 (0.47–0.88), p = 0.103, 11 cases and 26 controls. AUC for NfL 0.33 (0.00–0.67), for tau 0.24 (0.00–0.58), for S100B 0.71 (0.39–1.00) and for NSE 0.24 (0.00–0.54). b) 25, AUC = 0.77 (0.61–0.93), p<0.05, 7 cases, 29 controls. AUC for NfL 0.29 (0.00–0.61), for tau 0.38 (0.00–0.78), for S100B 0.38 /0.02–0.74) and for NSE 0.48 (0.12–0.84). c) 28, AUC = 0.75 (0.56–0.95), p<0.05, 7 cases, 27 controls. AUC for NfL 0.29 (0.00–0.64), for tau 0.05 (0.00–0.18), for S100B 0.45 (0.06–0.85) and for NSE 0.43 (0.05–0.81). d) 33, AUC = 0.89 (0.73–1.00), p<0.01, 6 cases, 28 controls. AUC for NfL 0.62 (0.17–1.00), for tau 0.57 (0.19–0.95), for S100B 0.95 (0.82–1.00) and for NSE 0.71 (0.37–1.00). e) 37, AUC = 0.83 (0.66–1.00), p<0.01, 8 cases, 24 controls. AUC for NfL 0.71 (0.24–1.00), for tau 0.31 (0.00–0.65), for S100B 0.69 (0.31–1.00) and for NSE 0.48 (0.06–0.89).

Area under the curve (AUC) in gestational week 10 was 0.67 (0.47–0.88) and in gestational week 25 the AUC was 0.77 (0.61–0.93). In gestational week 28, AUC was 0.75 (0.56–0.95) and in gestational week 33 AUC was 0.89 (0.73–1.00). In gestational week 37 the AUC was 0.83 (0.66–1.00). (Fig 3A–3E)

The combination of the four biomarkers was superior to the biomarkers alone or in combination of two or three (data not shown).

## Discussion

Plasma concentrations of both tau and NfL are increased in the end of pregnancy in women developing preeclampsia in contrast to healthy pregnancies. In the same cohort we have earlier shown that plasma concentrations of S100B and NSE are also increased in preeclampsia.[11, 12] Together, this supports that the central nervous system (CNS) is affected in preeclampsia early even in mild to moderate disease. When all four biomarkers of interest were analyzed together, AUC reached above 0.7 already from gestational week 25, indicating early CNS involvement months before onset of clinical disease. These findings emphasize the theory that preeclampsia begins early in gestation.

The strengths of this study are that it is a prospective cohort design, making the population less prone to bias, it is also a population well characterized concerning diagnosis of preeclampsia. Serial blood samples available throughout pregnancy make it possible to evaluate the biomarkers before onset of disease. There are also some limitations to the study. A fairly small number of women developed preeclampsia and the women diagnosed had preeclampsia without cerebral adverse events. The blood samples from healthy pregnant women were not from the entire cohort but only from a random sample of controls, limiting the interpretation of prediction from the results. The ROC curves constructed are based on few individuals and the models can be over-fitted and should therefore be interpreted with caution. A larger study needs to confirm these findings. The analyzes were done 9 years after collection of samples due to that the new ultrasensitive methos (Simoa) has not previously been available and there have been difficulties to analyze NfL and tau in peripheral blood.

To the best of our knowledge, there are no published data on long-term stability of tau or neurofilament light (NfL) in frozen blood samples. However, both proteins show long-term stability in CSF at both -20 and -80 oC and their concentrations are not affected by blood contamination.[19, 20] Further, we have unpublished data showing stability of tau and NfL concentrations in both plasma and serum over 4 freeze-thaw cycles (unpublished observation), suggesting that both proteins are quite stable in blood. If the concentrations would change slightly over time in frozen conditions, this would the case for both cases and controls, minimizing the potential error in difference between groups. Normally, proteins are stable in frozen conditions and these samples were just frozen once before analysis.

In this cohort, the diagnosis of preeclampsia was made on both hypertension and proteinuria. In the new definition by ISSHP from 2014,[21] preeclampsia diagnosis can be established without proteinuria if other signs of organ involvement exist. This study was conducted during a time period when proteinuria was still required for diagnosis. Also, ISSHP still recommends proteinuria as a requirement for preeclampsia diagnosis in the research setting to ensure more specificity around the diagnosis.[21]

Plasma concentrations of NfL and tau have been investigated extensively in Alzhemier’s disease and other CNS disorders[22–24] but have not yet been investigated in preeclampsia. For tau, the correlation between cerebrospinal fluid (CSF) and plasma concentrations is weak[18] wherefore other sources of origin (or other potential confounders) of the plasma tau signal have to be considered. Lederer et al showed that concentrations of tau and phospho-tau-181 in CSF did not differ between women with preeclampsia and normal pregnant controls at time of delivery in third trimester.[25] Our finding demonstrates that peripheral circulating concentrations of tau differ between groups. If one hypothesizes that CSF levels would not differ between groups also in our study, then the increased peripheral levels of tau could either be due to confounding sources outside the CNS or an increased secretion of tau into the extracellular space together with an altered blood brain barrier (BBB) that allows increased secretion of tau into the systemic circulation in preeclampsia. For NfL, the strong correlation of plasma with CSF NfL concentrations[15] and the lack of influence of BBB injury, as reflected by the CSF/serum albumin ratio, on serum NfL[26] suggest that the protein is mainly a marker of CNS neuronal injury. Considering that tau and NfL are predominantly localized in neurons in the CNS, the most likely explanation for their efflux into the blood would be that at least a certain degree of neuronal and/or BBB injury occurs even in preeclampsia without clinical signs of end organ involvement.

There are some promising models for prediction of preeclampsia already in the first trimester and also in the second trimester.[27] Some of the models predict early onset preeclampsia, others predict adverse outcome and time to delivery in women with already diagnosed preeclampsia.[2, 27] There is ongoing research to find a predictive model with or without biomarkers to predict maternal and neonatal complications in women diagnosed with preeclampsia. Studies exploring clinical predictors include the PREP, PIERS and PETRA cohorts and have mainly focused on early onset preeclampsia with populations consisting of 946, 636 and 216 women respectively with complications mainly of sorts other than neurological. The AUC for adverse outcome was about 0.80.[2, 28] A recent study investigating s-Flt-1/PlGF ratio in a low risk cohort of women evaluated the risk of severe preeclampsia at 36 gestational weeks with an AUC of 0.81. In this cohort, there was also a low rate of neurological complications, where 10/109 women with severe preeclampsia had visual disturbances and no woman had eclampsia.[29]

Cerebral biomarkers in preeclampsia have been investigated mainly with the hypothesis that they might be useful to avoid cerebral complications in women already diagnosed with preeclampsia.[11–13] Cerebral biomarkers can not be interpreted as possible predictors of onset of preeclampsia since such a biomarker has to have a high precision and sensitivity before onset of clinical disease. Though the fact that cerebral biomarkers in combination reach a fairly good diagnostic accuracy as early as in gestational week 25 is interesting from a pathophysiological perspective where the maternal brain in preeclampsia might be affected earlier than we previously thought. This study is not a diagnostic accuracy study but rather a pilot study where our findings need to be confirmed and explored in other patient populations.

For preeclampsia, with the evidence present today, it might be hard to predict all preeclampsia with one biomarker specifically for the disease since preeclampsia is an endothelial disease where different organ system are affected differently in different women and over different time periods during gestation.[27] The cerebral biomarkers might have a role either to predict early cerebral changes in pregnancy in women who later develop preeclampsia alone or in combination with other predictors and/or to predict the severity of the disease and potential cerebral complications once preeclampsia is diagnosed. This study must be considered as a pilot study and larger cohorts for prediction of preeclampsia or complications of preeclampsia are needed to confirm our findings.

A reliable cerebral biomarker in women with preeclampsia would be of use when allocating neuro protective treatment such as magnesium sulphate [30] to reduce the numbers needed to treat or when deciding to deliver or not, especially in the case of prematurity.[1]

Pathophysiological processes causing preeclampsia lead to endothelial injury and our results support a possible blood brain barrier compromise alone or in combination with neuronal and glial damage early in the course of disease. In this material, the source of the increased levels of the cerebral biomarkers cannot be confirmed.

## Supporting information

S1 Table Minimal data set. (XLSX)
